# *Bacillus amyloliquefaciens* LM-1 Affects Multiple Cell Biological Processes in *Magnaporthe oryzae* to Suppress Rice Blast

**DOI:** 10.3390/microorganisms12061246

**Published:** 2024-06-20

**Authors:** Meiling Liang, Aiqing Feng, Congying Wang, Xiaoyuan Zhu, Jing Su, Zihan Xu, Jianyuan Yang, Wenjuan Wang, Kailing Chen, Bing Chen, Xiaopeng Lin, Jinqi Feng, Shen Chen

**Affiliations:** 1 Guangdong Provincial Key Laboratory of High Technology for Plant Protection, Plant Protection Research Institute of Guangdong Academy of Agricultural Sciences, Guangzhou 510640, China; liangmeilingjane@163.com (M.L.); fengaq@gdppri.com (A.F.); adee117@163.com (C.W.); zhuxy@gdppri.com (X.Z.); bsujing@126.com (J.S.); yangjianyuan@gdaas.cn (J.Y.); juanlook@163.com (W.W.); 13128680287@163.com (K.C.); xx-man007@163.com (B.C.); lin810923464@163.com (X.L.); 13926108897@163.com (J.F.); 2 School of Life Sciences, South China Normal University, Guangzhou 510631, China; 2022023154@m.scnu.edu.cn

**Keywords:** *Magnaporthe oryzae*, *Bacillus amyloliquefaciens* LM-1, appressorium, cell death, autophagy

## Abstract

*Magnaporthe oryzae*, one of the most destructive rice pathogens, causes significant losses during the rice harvest every year. *Bacillus amyloliquefaciens* has been explored in many crops as a potential biocontrol agent. However, the mechanisms of *B. amyloliquefaciens* controled rice blast are not fully understood. Here, a biocontrol strain LM-1, isolated from a contaminated medium, was identified as *B. amyloliquefaciens* using morphological observation, physiological and biochemical tests, and 16S rDNA sequencing. LM-1 inhibited the growth and pathogenicity of *M. oryzae* and *Bipolaris oryzae* (Breda de Haan) Shoem. The mycelia of *M. oryzae* co-cultured with LM-1 were enlarged and broken by fluorescence microscopy using calcofluor white. LM-1 inhibited the mycelia of *M. oryzae* from producing conidia. Genes *itu*, *srf*, and *fenB* were detected in LM-1. Furthermore, the supernatant of LM-1 interfered with the appressorium formation of *M. oryzae*, blocked conidial cell death, and reduced autophagy degradation but did not affect the normal germination of rice seeds and seeding growth. Additionally, we observed hypersensitivity reactions, reactive oxygen species, and iron accumulation reduction in rice cells inoculated with supernatant. Our study reveals that LM-1 has a control effect on rice blast and affects cell wall integrity, sporulation, appressorium formation, cell death, and autophagy.

## 1. Introduction

*Magnaporthe oryzae*, which is listed among the top ten most important plant pathogens [1,2,3,4], poses a great threat to rice security. The formation of asexual conidia in *M. oryzae* requires exposure to light. The contents of conidia are degraded by autophagy to form the appressorium, a crucial structure that generates enormous turgor pressure to breach the waxy cuticle of the rice leaf [5]. Therefore, autophagy-dependent conidial cell death is essential for the maturation and infectivity of the appressorium [6]. Although chemical fungicides are a primary method for controlling the disease [7,8], they pose environmental risks and can lead to resistance. Thus, sustainable control methods need to be explored.

Biocontrol offers an effective and sustainable approach to protect rice yield while minimizing environmental damage [9,10]. Various *Bacillus* species, including *Bacillus amyloliquefaciens*, *Bacillus velezensis*, *Bacillus siamensis*, and *Bacillus subtilis*, have shown significant potential in reducing disease incidence across different hosts [11,12]. *B. amyloliquefaciens* is a gram-positive bacterium capable of producing starch hydrolase and a variety of secondary metabolites, such as surfactin, fengycin, and iturin, which exhibit strong antagonistic effects against bacterial and fungal pathogens [13,14,15]. Several strains of *B. amyloliquefaciens* have been reported to control plant pathogens effectively. For instance, strains MEP218 and ARP23 have shown biocontrol efficacy against sclerotinia stem rot disease through the production of cyclic lipopeptides iturin, surfactin, and fengycin [16]. Strain W10 has demonstrated significant biocontrol potential against *Rhizoctonia cerealis* [17]. Strain FZB42-AK3, which produces surfactin but not bacillomycin D and fengycin, significantly inhibits the pathogenicity of *M. oryzae* [18]. Additionally, strain UASBR9 not only suppresses *M. oryzae* but also promotes plant growth [14]. Although these reports focus on the antifungal activity and metabolites of *B. Amyloliquefaciens*, the specific mechanism of inhibition against *M. oryzae* remains unknown.

In this study, we investigated the mechanism of *B. amyloliquefaciens* LM-1 inhibiting the biological processes and pathogenicity of *M. oryzae*. Unlike previous studies where *B. amyloliquefaciens* was primarily isolated from host plants, our study is the first time a strain has been isolated from the air. The main objectives were to isolate and identify the antagonistic strain LM-1 against *M. oryzae*, evaluate its efficacy against rice diseases through pot experiments and tests on detached leaves, and assess whether the biological processes of *M. oryzae* are inhibited by *B. amyloliquefaciens* LM-1. These results provide a theoretical foundation for developing biological control agents for the comprehensive treatment of rice blast and other rice fungi.

## 2. Materials and Methods

### 2.1. Strains and Growth Conditions

The wild-type B157, GFP-ATG8 (autophagy marker), hH1-GFP (cell nuclear marker), and Cyto-GFP (cytoplasmic marker) strains of *M. oryzae* were used in this study [19,20]. Strain LM-1 was isolated from the contaminated Prune agar (PA) culture medium with *M. oryzae* and inoculated on Luria–Bertani (LB, tryptone 10 g/L, yeast extract 5 g/L, NaCl 10 g/L, agar 13 g/L) agar plates at 28 °C for 2 days. The *M. oryzae* B157 strain and *Bipolaris oryzae* (Breda de Haan) Shoem were cultivated on PA medium [19,21] at 28 °C, for 4 days in the dark, followed by 12 h (h) light/dark cycle for 4 days to induce conidiation.

To prepare the fermentation broth (FTB), supernatant (SUP), and bacterial sediment (BTS), *B. amyloliquefaciens* LM-1 was inoculated in LB liquid medium (tryptone 10 g/L, yeast extract 5 g/L, NaCl 10 g/L). The mother liquor was obtained by shaking the culture at 28 °C and 200 rpm overnight. Then, 1% of the mother liquor of strain LM-1 was inoculated in LB liquid medium under the same conditions for 6–8 h to obtain OD 600 = 1.0 FTB. The FTB was centrifuged at 12,000 rpm for 15 min, and the SUP and BTS were collected. The SUP was filtered through a 0.22 μm biofilter and the BTS was diluted with sterile water.

### 2.2. Identification of B. amyloliquefaciens LM-1

Genomic DNA extraction was carried out using the Omega Bacterial DNA Kit (D3350-01). The primers for 16S rDNA sequence of strain LM-1 were 27F (5′-AGAGTTTGATCCTGGCTCAG-3′) and 1492R (5′-GGTTACCTTGTTACGACTT-3′). The 16S rDNA sequence of LM-1 predicted by BLAST was compared with NCBI, with a parameter identify >95. The top 12 16S rDNA sequences with the highest identify were selected. The 16S rDNA sequence of LM-1 and 12 related *Bacillus* species (*Bacillus amyloliquefaciens* strains: PP19, 205, YP6, and 97; *Bacillus velezensis* strains: CACC 316, Bac57, DKU_NT_04, and Lzh-a42; *Bacillus sp.* strains: L381, Lzh-5, BH072, and YBsi01) were used to construct a phylogenetic tree with MEGA 7.0 using the Neighbor-Joining method and Muscle software (https://drive5.com/muscle/ accessed on 15 June 2024) for multiple sequence alignment.

### 2.3. Enzyme Activity Detection

Enzyme activity detection included amylase (Aml), protease (Prt), cellulase (Cel), and pectate lyase (Pel) activities of LM-1, which were detected on agar plates containing starch, skim milk, sodium carboxymethyl cellulose, and polygalacturonan, respectively [22,23,24]. The LM-1 suspension (2 μL, OD 600 = 1.0) was inoculated on four testing media plates and cultured at 28 °C for 2–3 days.

### 2.4. Genome Annotation of B. amyloliquefaciens LM-1

Genome sequencing was completed by Sangon Biotech in Guangzhou. The raw sequencing data were evaluated using FastQC. Ribosomal RNA and tRNA genes were predicted by Prokka. The complete genome of strain LM-1 was visualized using Circos. Whole genome BLAST searches were performed with NR, COG, SwissProt, CDD, KOG, NT, PFAM, and TrEMBL to obtain functional annotation information. GO function annotation was obtained based on SwissProt and TrEMBL annotations, while KEGG annotation was obtained using KAAS.

### 2.5. Sensitivity Detection for Mycelial Growth of M. oryzae and B. oryzae with B. amyloliquefaciens LM-1

The FTB concentration of *B. amyloliquefaciens* LM-1 was adjusted to OD 600 = 1.0. PA medium (20 mL) was placed in a 9 cm petri dish. After cooling, the colony mass of *M. oryzae* and *B. oryzae* was placed in the middle of the PA plate, respectively. The FTB was dripped onto three sterile circular filter papers around the colony mass of *M. oryzae* and *B. oryzae*. The plates were co-cultured in a 28 °C incubator for 7 days in the dark. The experiment was repeated three times.

### 2.6. Pathogenicity Detection

For spray inoculation of rice seedlings, four treatments of 2-week-old rice seedlings (CO39) were sprayed with conidial suspension (1 × 10^5^ conidia/mL, 20 mL per pot with ten seedlings) of *M. oryzae* and *B. oryzae* and kept in a growth chamber at 28 °C with 90% humidity for 24 h in the dark, followed by a 12 h light/12 h dark cycle. The treatments included: (1) rice seedlings sprayed with conidial suspensions of *M. oryzae* B157; (2) rice seedlings sprayed with conidial suspensions of *B. oryzae*; (3) rice seedlings sprayed with a mixture of conidial suspensions of *M. oryzae* B157 and the FTB of LM-1; (4) rice seedlings sprayed with a mixture of conidial suspensions of *B. oryzae* and the FTB. The severity of rice leaf lesions caused by *M. oryzae* and *B. oryzae* was quantified according to the standard evaluation system for leaf spots [19,21]. The grades of lesion severity were based on the percentage of leaf area affected by lesions. The proportion of rice leaves with different grades was calculated [19,21]. Leaf lesions were photographed at 7 days post-inoculation.

Rice leaf explants infection assays were performed by inoculating 2-week-old detached rice leaves (4–6 cm per leaf) with conidial suspensions (1 × 10^5^ conidia/mL, 3–5 drips of 20 μL per drip) of *M. oryzae* treated with or without the FTB, BTS, and SUP of strain LM-1 at different time points. The treatments included: (1) detached rice leaves incubated with conidial suspensions of *M. oryzae* B157; (2) detached rice leaves incubated with the FTB, BTC, and SUP of strain LM-1, respectively, with water as a negative control (CK); (3) 20 μL of the FTB, BTS, and SUP of strain LM-1, respectively, dripped onto detached rice leaves after the strain B157 treatment at 24 h (−24 h); (4) 20 μL of the FTB, BTS, and SUP of strain LM-1, respectively, dripped onto detached rice leaves before the strain B157 treatment at 24 h (+24 h); (5) detached rice leaves incubated with conidial suspensions of B157 mixed with FTB, BTS, and SUP of strain LM-1, respectively (0 h). These treatments were kept at 28 °C with 90% humidity for 24 h in a dark incubator, followed by a 12 h light/12 h dark cycle. Leaf lesions were observed at 7 days post-inoculation. The experiments were repeated three times.

Leaf sheaths were removed from 2-week-old rice seedlings and inoculated with conidial suspensions (1 × 10^5^ conidia/mL) of strain Cyto-GFP with or without the SUP of *B. amyloliquefaciens* LM-1 for sheath infection assays, incubated in dark and humid conditions for 48 h. The infection hyphae of *M. oryzae* were observed and photographed with a fluorescence microscope (Zeiss, AxioScope A1, Jena, Germany). Three biological repeats were performed.

### 2.7. Microscopy Observation

The morphology of strain LM-1 was observed with a transmission electron microscope (TEM, Hitachi, HT7700, Tokyo, Japan). After overnight culture in LB liquid medium at 200 rpm 28 °C, the concentration of strain LM-1 was adjusted to OD 600 = 1.0, and 2.5% glutaraldehyde fixing solution was added at room temperature.

The FTB concentration of strain LM-1 was adjusted to OD 600 = 1.0. PA (20 mL) medium was placed in a 9 cm petri dish. After cooling, the colony mass of *M. oryzae* and *B. oryzae* were placed in the middle of the PA plate, respectively. The FTB was dripped onto three sterile circular filter papers around the colony mass of *M. oryzae* and *B. oryzae*. The plates were cultured at 28 °C, in the dark for 3 days, followed by a 12 h light/dark cycle for 3 days to induce conidiation, and observed with a microscope (Leica, DM500, Wetzlar, Germany).

To observe the hypersensitivity reaction (HR), reactive oxygen species (ROS), and ferric ion (Fe^3+^) in rice leaf sheath cells, the rice leaf sheaths of 2-week-old seedlings were inoculated with *M. oryzae* conidial suspension with or without SUP of *B. amyloliquefaciens* LM-1, kept at 28 °C in a dark incubator for 48 h. DAB and Prussian blue staining for visualization of ROS and Fe^3+^ were performed according to established methods [19,25]. The HR, ROS, and Fe^3+^ signals were observed with a microscope (Leica, DM500).

Conidial viability was evaluated by examining intact nuclei, visualized by the Histone H1-GFP reporter in the wild type B157 strain [19]. To observe appressorium formation and cell nuclei, 20 μL of the mixture of the SUP of strain LM-1 and the conidia suspension of strain hH1-GFP were dripped onto the surface of the hydrophobic glass coverslips and incubated at 28 °C for 2, 8, and 24 h. A conidial suspension without the SUP of strain LM-1 was used as a control. For appressorium formation and cell nuclei, the fluorescence microscope AxioScope A1 (Zeiss, Germany) was used with the GFP channel (488 nm). This experiment was repeated three times.

Autophagy was examined by the GFP-Atg8 reporter in the wild type B157 strain [19]. The germination tubes and appressoria were observed under a fluorescence microscope AxioScope A1 (Zeiss, Germany) after incubating conidia with or without the SUP of *B. amyloliquefaciens* LM-1 on the hydrophobic glass coverslips at 2 and 6 h. For hH1-GFP or GFP-Atg8, the fluorescence microscope was used with the GFP channel (488 nm).

Calcofluor white (CFW, 10 μg/mL, Sigma-Aldrich) staining was used to observe hyphal with or without the SUP of *B. amyloliquefaciens* LM-1. The mycelia of *M. oryzae* were co-cultured with the SUP in complete medium (CM, yeast extract 6 g/L, casein hydrolysate 6 g/L, sugar 10 g/L, pH 6.5) liquid medium at 28 °C with a shaker 200 rpm for 2 days. The sample was then placed on a slide, a drop of CFW and 10% potassium hydroxide was applied, and the cover glass was placed over the sample to stain 3–5 min. Finally, the samples were observed under the fluorescence microscope with ultraviolet light (Zeiss, Germany).

### 2.8. Effects of Strain LM-1 on Growth and Development of Rice

The rice seeds (CO39) with full grains were selected. The seeds were disinfected with 75% ethanol for 1 min and then washed several times with water. After surface disinfection, 100 rice seeds were soaked in water and the SUB of LM-1 strain, respectively, for 10 h, and then washed three times with clean water. After incubating at 28 °C for 48 h, the germination rate was calculated. Seeds with the same germination were selected and cultured in 28 °C (light 16 h/dark 8 h) in water and SUB for 7 days, respectively. The root length and sprout length of rice were measured. Each treatment group had 10 rice seedlings. All experiments were repeated three times.

## 3. Results

### 3.1. Isolation and Identification of Bacillus amyloliquefaciens LM-1

**Figure 1:**
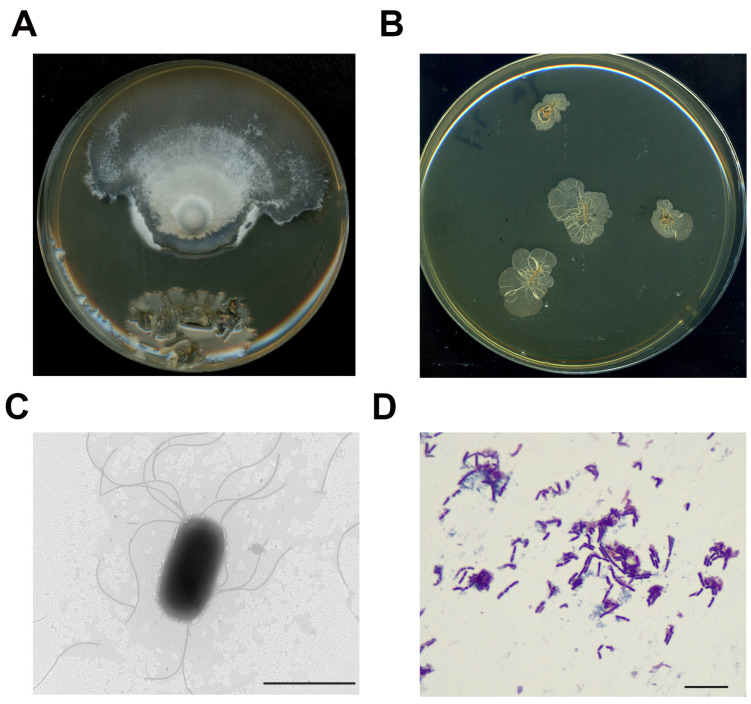
Isolation and identification of *B. amyloliquefaciens* LM-1. (**A**) *M. oryzae* + LM-1 were co-cultured on PA medium. (**B**) LM-1 was cultured on LB medium. (**C**) LM-1 was observed by transmission electron microscope (TEM). Scale bar = 2.0 µm. (**D**) LM-1 was dyed by gram staining. Scale bar = 20 µm.

The strain LM-1 was isolated from a contamination PA plate of *M. oryzae* (Figure 1A), where it inhibited the mycelium growth of *M. oryzae*, and was selected for further study. When cultured on LB medium, LM-1 formed opaque colonies with surface folds and irregular edges (Figure 1B). Observations under a transmission electron microscope (TEM) and a general optical microscope revealed rod-like cells with multiple flagella (Figure 1C). Gram staining indicated that LM-1 was a gram-positive bacterium (Figure 1D). Thus, strain LM-1 is identified as *Bacillus* species.

**Figure 2:**
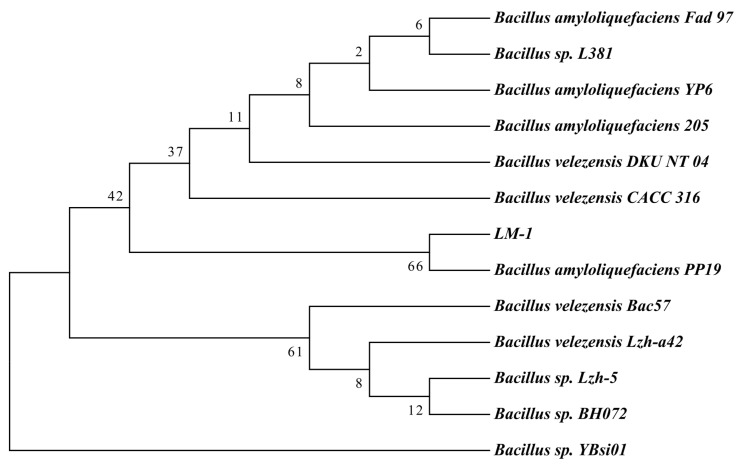
The phylogenetic tree of strain LM-1 with its close relatives *Bacillus* species based on 16S rDNA gene sequencing. Neighbor-Joining phylogenetic tree of the strain LM-1 was constructed by MEGA 7.0. The phylogenetic tree of *B. amyloliquefaciens* LM-1 and 12 other *Bacillus* species were based on 16S rDNA sequence analysis. The numbers at the branches indicate the confidence level calculated by bootstrap analysis (1000).

The 16S rDNA gene sequences of LM-1 were amplified by PCR (Figure S1A). A phylogenetic tree constructed with LM-1 and 12 other *Bacillus* species showed that LM-1 was closely related to *Bacillus amyloliquefaciens* PP19 (Figure 2). For comparing with 16S rDNA gene sequence in the NCBI database, strain LM-1 showed 99.7% similarity with *B. amyloliquefaciens* PP19 and was identified as *B. amyloliquefaciens*. Based on all evidence mentioned above, strain LM-1 is classified as *B. amyloliquefaciens*.

### 3.2. Genomic Features of B. amyloliquefaciens LM-1

**Figure 3:**
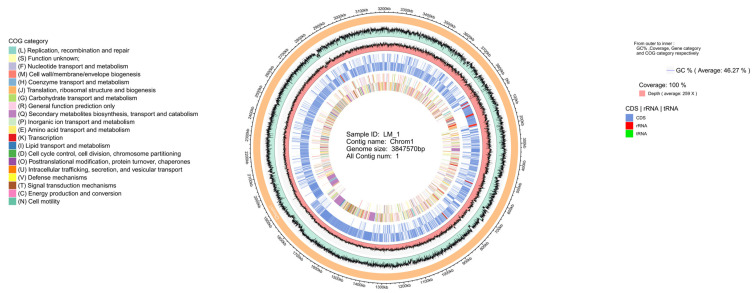
Genomic Features of *B. amyloliquefaciens* LM-1. Circular genome map of *B. amyloliquefaciens* LM-1 was shown from the outside to the inside, GC content, sequencing depth, gene elements and COG functions.

The complete genome sequence of *B. amyloliquefaciens* LM-1 was composed of a circular chromosome of 3,847,570 bp (Figure 3), and displayed by Circos software (BLAST Ring Image Generator download|SourceForge.net), including GC content, sequencing depth, gene element content, and COG functions. The genome included 3897 protein-coding genes with an average length of 889.17 bp, ranging from 57 bp to 16,314 bp, total coding gene was 3,465,077 bp, with a coding ratio of 90.06%, and an average G+C content of 46.27% (Dataset S1). The chromosome also contained 86 tRNA and 27 rRNA (Dataset S1). Genes were classified by GO into biological process, cellular component, and molecular function (Figure S2). KEGG pathways analysis revealed five branches: cellular processes, environmental information processing, genetic information processing, metabolism, and organismal systems (Figure S3). Among the predicted genes, 3688 (99.59%), 3477 (93.90%), 2787 (75.26%), and 1633 (44.10%) matched the NR, SwissProt, COG, and KEGG databases, respectively (Dataset S2, Figure S4). A total of 1594 genes matched across all four databases (Figure S5).

### 3.3. Enzyme Activity Detection of B. amyloliquefaciens LM-1

**Figure 4:**
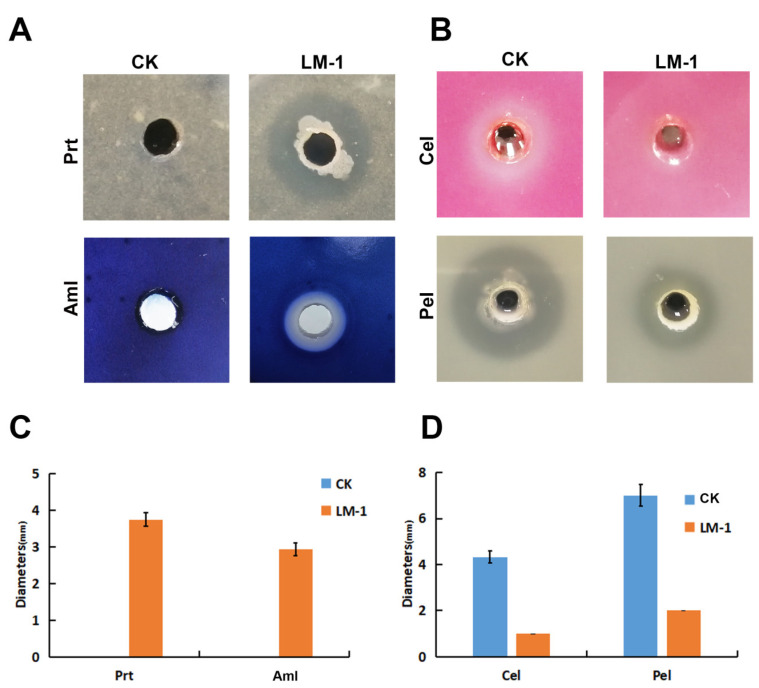
Enzyme activity detection of *B. amyloliquefaciens* LM-1. (**A**,**C**): amylase (Aml) and protease (Prt) production detection of bioassay plate, the statistics of Aml and Prt degradation diameter. LB liquid medium served as a negative control (CK). (**B**,**D**) cellulase (Cel) and pectate lyase (Pel) production detection of bioassay plate, the statistics of Cel and Pel degradation diameter. *Dickeya zeae* EC1 was used as a positive control (CK).

To clarify the enzyme activity of *B. amyloliquefaciens* LM-1, we tested its amylase (Aml), protease (Prt), cellulase (Cel), and pectate lyase (Pel) activities. Compared to the control group, LM-1 showed significantly larger zona pellucida diameter for Aml and Prt (Figure 4A,C), indicating its ability to degrade starch and protein, consistent with *B. amyloliquefaciens*. *Dickeya zeae* EC1 was used as a positive control for Cel and Pel activities [26]. LM-1 showed no activity in the Cel medium and only slight activity in the Pel medium compared to EC1 (Figure 4B,D). Thus, LM-1 possesses Aml, Prt, and Pel but not Cel activity.

### 3.4. The Rice Fungal Diseases Can Be Controlled by B. amyloliquefaciens LM-1

**Figure 5:**
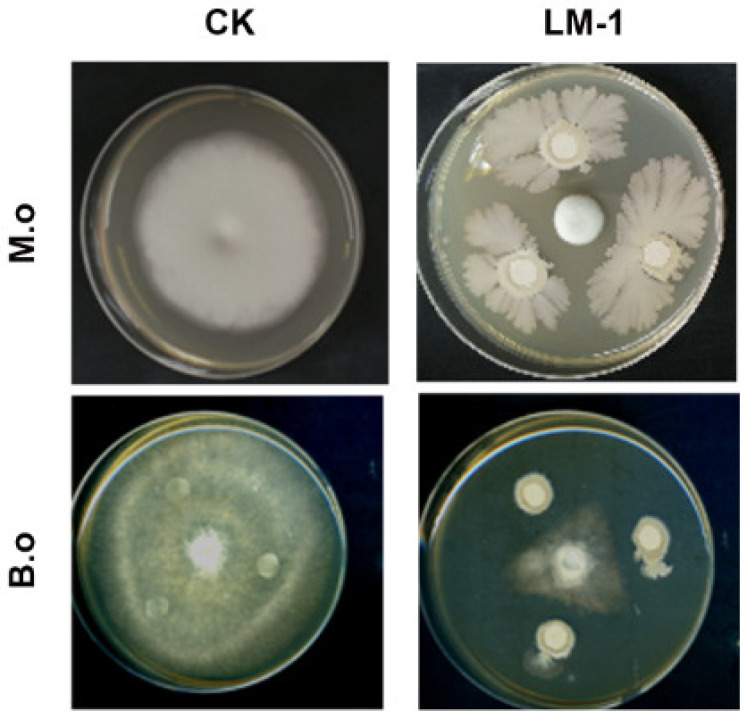
The Antifungal activity test between LM-1 and fungal pathogens. Fungal plate photographs were taken after 7 days. All plates were placed at 28 °C. *M. o*: *M. oryzae*, *B. o*: *B. oryzae*.

To determine the broad-spectrum resistance of LM-1, its fermentation broth (FTB) was used in an antagonistic experiment. It was found that *M. oryzae* and *B. oryzae* can be inhibited by the FTB (Figure 5). In rice seedlings infection assays, the rice leaves of control groups were susceptible to pathogenic fungi at 7 days post-inoculation (Figure S6). The proportions of grades in 6, 7, and 8 were 43.3%, 43.3%, and 13.3% in *M. oryzae*; grades of 7 and 8 were 36.7% and 43.3 % in *B. oryzae* (Table 1). In treatment group *M. oryzae* with FTB, the proportions of grades in 1, 2, and 3 were 50.0%, 30.0%, and 20.0%, while grades of 4, 5, and 6 were 33.3%, 30.0%, and 26.7% in *B. oryzae* with FTB (Table 1). Therefore, *M. oryzae* and *B. oryzae* were effectively inhibited by strain LM-1, a significant reduction in the lesions, particularly against *M. oryzae*. Overall, LM-1 shows protective and curative effects on rice against fungal diseases, with the highest biocontrol efficiency against *M. oryzae*.

**Figure 6:**
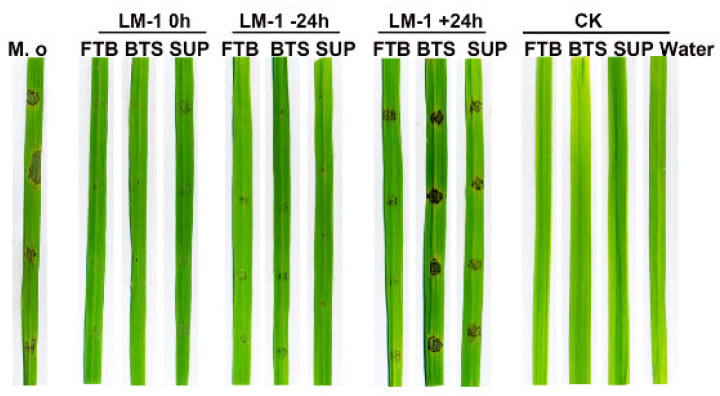
The biocontrol efficacy of strain LM-1 on rice blast. Pathogenesis assay with detached rice leaves (CO39). FTB, BTS, and SUP. were added to conidial suspension concurrently (0 h), 24 h before (−24 h), or after (+24 h) conidia inoculation on detached rice leaves. Detached rice leaves were incubated with the FTB, BTC, and SUP, respectively, with water as a negative control (CK). Images were taken at 7 days.

PCR analysis detected antimicrobial peptide biosynthetic genes *(srf*, *itu*, *and fen)* in LM-1 (Figure S1B). To analyze these metabolites’ roles, we tested rice leaves inoculated with LM-1’s FTB, SUP, and BTS under simultaneous inoculation (0 h), preventive inoculation before 24 h (−24 h), and inoculation after 24 h (+24 h). The effect of LM-1’s FTB, SUP, and BTS in simultaneous inoculation and preventive inoculation before 24 h were more significant than the control group, and the lesion was significantly weakened (Figure 6). The FTB had more significant antagonistic activity against *M. oryzae* than SUP and BTS in +24 h (Figure 6). The results show that the FTB, SUP, and BTS of *B. amyloliquefaciens* LM-1 have significant antagonistic activity against *M. oryzae*, especially FTB.

**Figure 7:**
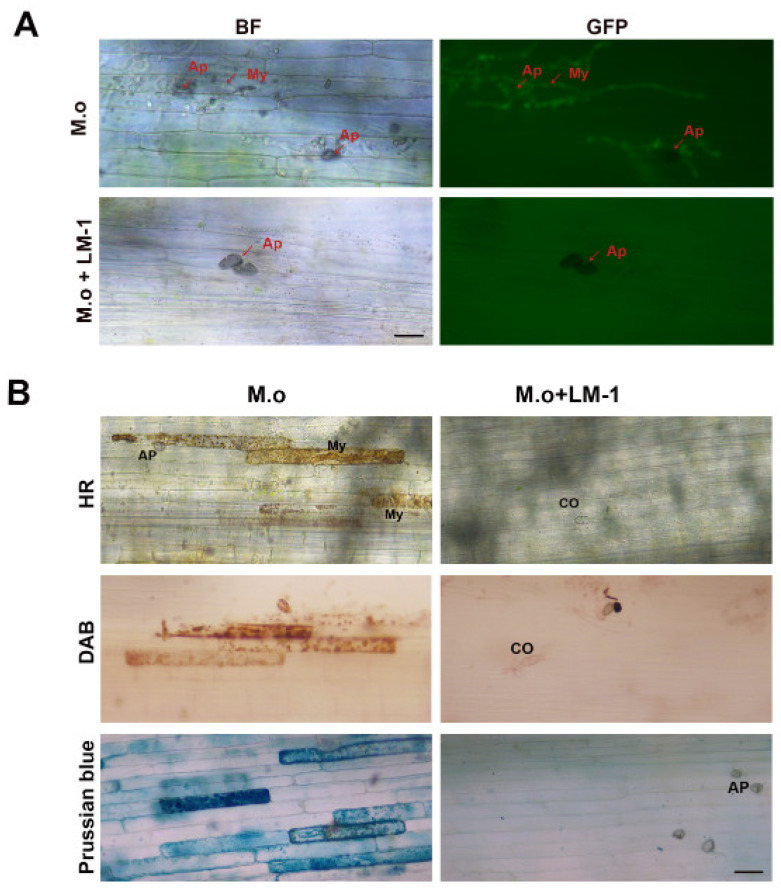
LM-1 inhibits the infection in the rice leaf sheath. (**A**) Effect of strain LM-1 on rice sheath inoculation during *M. oryzae* infection. *M. o*: suspension concentration 10^5^ conidia/mL in water; *M. o* + LM-1: suspension concentration 10^5^ conidia/mL with the SUP of LM-1; BF: bright field; GFP: green fluorescent protein. Ap: appressorium. My: mycelium. Scale bar = 10 µm. (**B**) Effect of LM-1 on rice HR, ROS, and Fe^3+^ accumulation during *M. oryzae* infection. HR, DAB, and Prussian blue staining were performed on the conidia-inoculated rice leaf sheath at 48 h. *M. o*: suspension concentration 10^5^ conidia/mL in water; *M. o* + LM-1: suspension concentration 10^5^ conidia/mL with the SUP of LM-1. Ap: appressorium. My: mycelium. CO: conidium. Scale bar = 10 µm.

After *M. oryzae* infected rice leaf sheath cells, the infection mycelium of strain Cyto-GFP in the control group spread from the initial infection site to peripheral cells (Figure 7A), showing normal invasion ability. Under the treatment of the SUP of LM-1, the infection mycelia of strain Cyto-GFP significantly decreased invasive growth of the pathogen compared with the control group at 48 h (Figure 7A), indicating that the SUP of LM-1 could significantly inhibit the *M. oryzae* from infecting plant cells.

To assess the effect of the SUP of LM-1 on rice, we next detected the hypersensitive response (HR) in rice leaf sheaths with SUP-conidial inoculation, stained with 3,3′-diaminobenzidine (DAB) and Prussian blue in treated leaf sheaths for to visualize ROS and ferric ions (Fe^3+^). We observed that the accumulation of HR, ROS, and Fe^3+^ sharply decline in rice leaf sheath cells inoculated with SUP-treated conidia compared to only conidia (Figure 7B). This may reflect a block of ROS and iron accumulation by LM-1 in rice cells.

### 3.5. LM-1 Inhibits Sporulation and Damages the Integrity of the Cell Wall in M. oryzae

**Figure 8:**
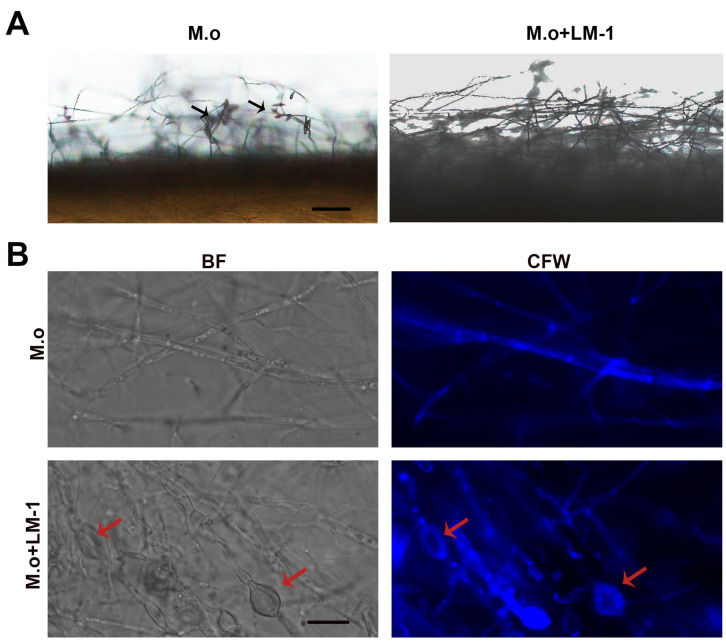
LM-1 inhibits sporulation and damages the integrity of the cell wall in *M. oryzae.* (**A**) LM-1 inhibited sporulation of *M. oryzae*. *M. o*: suspension concentration 10^5^ conidia/mL in water; *M. o* + LM-1: suspension concentration 10^5^ conidia/mL with the SUP of LM-1. The black arrows indicated conidia. Scale bar = 20 μm. (**B**) LM-1 destroyed cell wall integrity of *M. oryzae*. LM-1 altered the distribution of chitin in the cell wall of *M. oryzae*. Hyphae were stained with 10 μg/mL calcofluor white (CFW) for 5 min in dark before being photographed. BF: bright field. The red arrow indicated mycelium enlargement. Scale bar = 10 μm.

To gain insights into the effect of LM-1 on asexual reproduction of *M. oryzae*, we monitored conidial production with an optical microscope. The mycelia of *M. oryzae* caused a serious reduction in conidiophore production after co-culture with the SUP of LM-1 (Figure 8A). CFW staining revealed swollen mycelia in *M. oryzae* treated with LM-1, indicating disrupted cell wall integrity (Figure 8B). Taken together, these results demonstrate that treatment with LM-1 interferes with the conidiophore production and the cell wall of *M. oryzae*.

### 3.6. LM-1 Suppresses Appressorium Formation and Conidial Cell Death of M. oryzae

**Figure 9:**
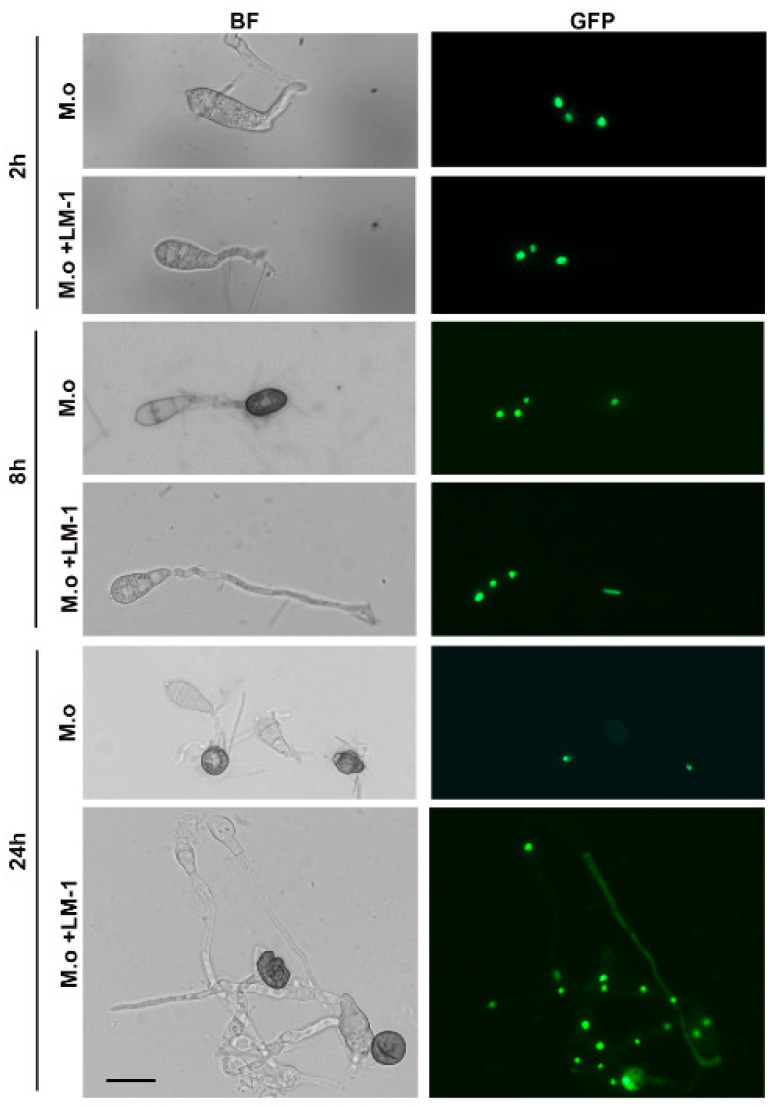
*B. amyloliquefaciens* LM-1 suppresses appressorium formation and conidial cell death of *M. oryzae.* Live cell imaging experiment to show strain hH1-GFP localization during *M. oryzae* appressorium development (2, 8, and 24 h) with or without the SUP of LM-1. *M. o*: suspension concentration 10^5^ conidia/mL in water; *M. o* + LM-1: suspension concentration 10^5^ conidia/mL with the SUP of LM-1. BF: bright field. GFP: green fluorescent protein. Scale bar = 10 μm.

To investigate the effects of LM-1 on conidial development, appressorium formation, and conidial cell death, we used strain h1H-GFP, a fluorescent nucleus marker, to visualize conidial cell death. At 2 h, conidial cells in both control and treated groups were alive with three nuclei (Figure 9). After 8 h, nuclei transferred to appressoria in the control group, but the conidia could not form appressorium normally, and nuclei remained in the germinated tube of the treatment group (Figure 9). At 24 h, the control group conidia had no nuclear GFP, only one nucleus in appressorium. However, the SUP of strain LM-1 treatment delayed conidial cell viability, and several nuclei appeared in the deformed tube and appressoria at 24 h (Figure 9). Taken together, we conclude that LM-1 inhibits conidial cell death during appressorium formation in *M. oryzae*.

### 3.7. Effects of LM-1 on Autophagy during Conidial Germination in M. oryzae

**Figure 10:**
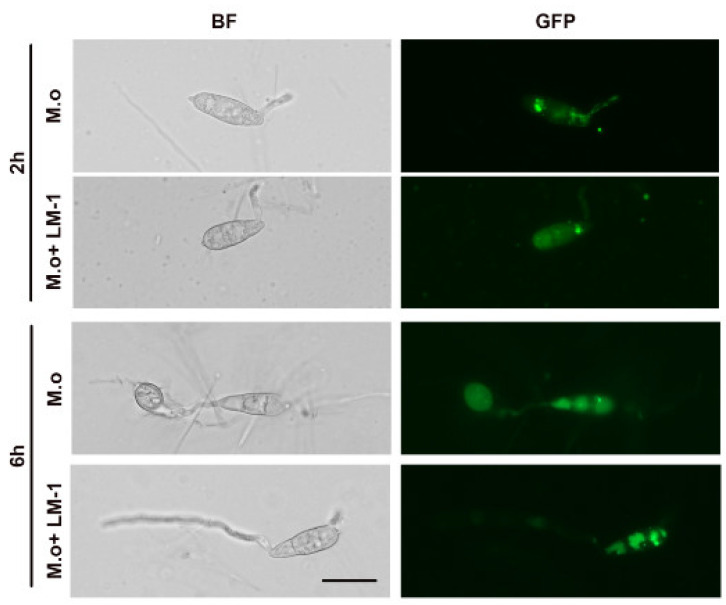
Effects of *B. amyloliquefaciens* LM-1 on *M. oryzae* autophagy. Epifluorescence images showing subcellular localization of GFP-Atg8 with or without the SUP of LM-1 during conidial development (2 and 6 h) are representatives of three biological replicates of the experiment. BF: bright field, GFP: green fluorescent protein. Scale bar = 10 μm.

To assess the effects of LM-1 on autophagy during conidial germination, strain GFP-Atg8 was used as an autophagic vesicle marker. At 2 h, numerous vesicular punctae (autophagosomes) were observed during conidial germination with or without the SUP of LM-1 (Figure 10). In the control group, GFP-Atg8 accumulated within large, spherical vacuoles in conidial cells at 6 h (Figure 10). Treatment with the SUP of LM-1 caused some punctate GFP-Atg8 (autophagosomes) to accumulate in the cytosol outside the vacuoles (Figure 10). We confirmed that the SUP of LM-1 treatment delayed the fusion of autophagosomes, which may also contribute to its suppression of conidial cell death in *M. oryzae.* Overall, LM-1 treatment affects autophagic activity in developing conidia.

### 3.8. Effects of Strain LM-1 on Growth and Development of Rice

**Figure 11:**
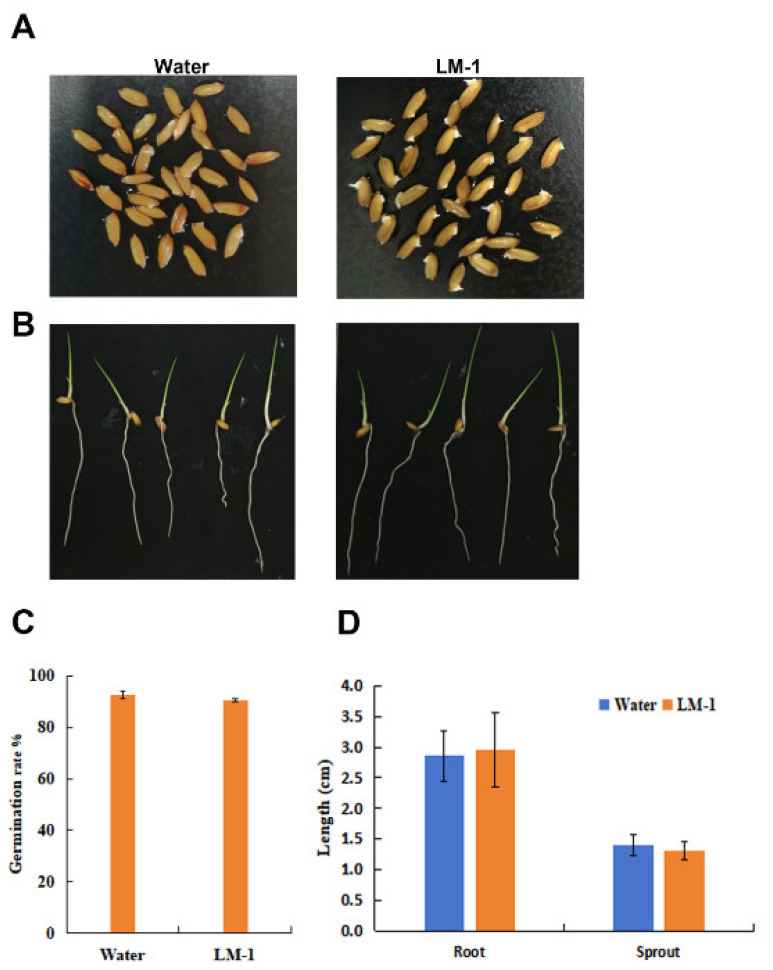
The germination of rice seeds and plant growth were not affected by *B. amyloliquefaciens* LM-1. (**A**) Rice seed germination. (**B**) The growth of rice root and sprout, Water: water treatment, LM-1: *B. amyloliquefaciens* LM-1. (**C**,**D**) the data for the rice germination rate%, and the length of rice root and sprout.

To determine whether LM-1 affected rice growth, the germination experiment with rice seeds (CO39) was conducted with the SUP of strain LM-1. The germination rate was unaffected by the SUP (Figure 11A,C), similar to water treatment. During rice seedling growth, the SUP of LM-1 was added to the culture medium. LM-1 did not affect the development of roots and sprouts compared to the control group (Figure 11B,D). Therefore, *B. amyloliquefaciens* LM-1 does not affect the normal germination or growth of rice seeds.

## 4. Discussion

*M. oryzae*, which occurs worldwide in rice and is difficult to manage [27,28], can infect the rice leaves, internodes, necks, spikes, and panicles. It causes economic losses due to rice yield loss. Therefore, controlling rice blast caused by this fungus is essential. Over the years, biocontrol has emerged as an effective, safe, and environmentally friendly method for plant disease management, reducing the reliance on synthetic agrochemicals [29,30]. *B. amyloliquefaciens*, a species within the *Bacillus* genus, has shown potential as a biocontrol agent against plant pathogens [31,32,33]. Previous studies have reported that *B. amyloliquefaciens* can effectively control rice blast [18,34], but the mechanisms underlying its inhibition of *M. oryzae* remain unclear.

In our study, a bacteria strain named LM-1 was isolated from contaminated media containing *M. oryzae* and exhibited significant antifungal activity against this fungus. We preliminarily identified strain LM-1 as *B. amyloliquefaciens* through morphological observation, physiological and biochemical tests, and 16S rDNA sequence. To further elucidate its characteristics, we performed whole genome sequencing and enzyme activity detection. Through confrontation and pot experiments, we demonstrated that *B. amyloliquefaciens* LM-1 can inhibit the growth and control the pathogenicity of both *M. oryzae* and *B. oryzae*, with a more pronounced effect on *M. oryzae*. These findings align with previous reports [18,34]. *B. amyloliquefaciens* produces surfactin, fengycin, and iturin, which have strong antagonism effects on bacterial and fungal plant pathogens [15,16,35], and our results confirmed these antagonistic properties.

To further substantiate these findings, we demonstrated that the SUP of *B. amyloliquefaciens* LM-1 not only inhibits appressorium formation and cell death but also affects the degradation of autophagosomes in *M. oryzae*. An intact cell wall in *M. oryzae* is essential for maintaining turgor pressure to penetrate the rice leaf epidermis [36]. Previous studies have shown that *Bacillus subtilis* can reduce cell wall integrity and appressorium formation in *M. oryzae* to control rice blast disease [37,38]. In our study, we found that *B. amyloliquefaciens* LM-1 reduced cell wall integrity in *M. oryzae* and significantly decreased appressorium formation on hydrophobic slides. In the leaf sheath inoculation experiment, the SUP inhibited invasive hyphal development. Conidial cell death is necessary for *M. oryzae* to infect the host [6,39]. We observed that cell death in *M. oryzae* was inhibited by the SUP of *B. amyloliquefaciens* LM-1, with conidia retaining multiple nuclei and malformed mycelia, unlike the untreated group where conidia degraded into a single nucleus in appressorium after 24 h.

The autophagy process ensures the degradation and recycling of conidial contents into the appressorium formation to form turgor [6,40,41]. To explore the relationship between SUP treatment and autophagy activity in *M. oryzae*, we tested the SUP and conidia of strain GFP-Atg8 on hydrophobic slides at 2 and 6 h. The degradation of the autophagosome in strain GFP-Atg8 was significantly delayed by SUP treatment at 6 h, indicating that the autophagy process in *M. oryzae* was decelerated under SUP stress. In previous reports, the autophagy of the hypha *M. oryzae* was impaired by *Bacillus subtilis* [37]. Omics data analysis revealed that the inhibition of *M. oryzae* by *Bacillus velezensis* downregulated the expression of genes involved in autophagy [42].

Numerous studies have revealed the effects of biocontrol bacteria on plant resistance. *Bacillus subtilis*, *Bacillus vallismortis*, and *Bacillus cereus* have been shown to inhibit rice blast by inducing plant resistance [43,44,45]. In our study, LM-1 did not affect the normal germination of rice seeds or the growth of rice seedlings. The biocontrol bacterium not only controls rice diseases but also does not affect the growth and development of rice, which is a crucial feature for its wide application. Furthermore, we observed that the HR, ROS, and iron accumulation were reduced in rice leaf sheath cells inoculated with SUP-treated conidia. Fungal infection induces the accumulation of ROS and iron, which triggers host HR or cell death [46,47]. SUP-treated conidia may not trigger HR in plants, blocking ROS and iron accumulation in rice cells. Overall, while LM-1 did not affect rice growth, it inhibits the pathogenicity of *M. oryzae*.

## 5. Conclusions

In conclusion, the novel strain *B. amyloliquefaciens* LM-1 has no obvious harmful effects on plants and would be an ideal biocontrol bacterium for controlling rice blast and other rice fungal diseases. *M. oryzae* could be controlled by the SUP of *B. amyloliquefaciens* LM-1 through the inhibition of appressorium formation, conidial cell death, and delayed autophagy degradation. The cell biological process changes in *M. oryzae* exposed to *B. amyloliquefaciens* LM-1 are the molecular basis for understanding the interaction between biocontrol bacteria and fungi. *B. amyloliquefaciens* LM-1 may as a possible natural biocontrol for rice blast.

## Figures and Tables

**Table 1:** Biocontrol efficacy of LM-1 on *M. oryzae and B. oryzae*.

Treatment Group	Disease Grade %							
	1	2	3	4	5	6	7	8
M.o						43.3% ± 9.81	43.3% ± 7.2	13.3% ± 2.72
M.o+LM-1	50.0% ± 4.7	30.0% ± 4.7	20.0% ± 4.7					
B.o					10.0% ± 8.2	10.0% ± 8.2	36.7% ± 9.8	43.3% ± 16.6
B.o+LM-1				33.3% ± 5.4	30.0% ± 8.2	26.7% ± 10.9	6.7% ± 5.4	3.3% ± 2.7

## Data Availability

The original contributions presented in the study are included in the article, further inquiries can be directed to the corresponding author.

## Supplementary Materials

The following supporting information can be downloaded at: https://www.mdpi.com/article/10.3390/microorganisms12061246/s1, Figure S1: Detection of 16S rDNA and antimicrobial peptide biosynthetic gene from *B. amyloliquefaciens* LM-1. (A) 16S rDNA from *B. amyloliquefaciens* LM-1 was amplified using the 27F/1492R primer. M: 2000 bp DNA marker; 1-3: 16S rDNA of LM-1. (B) Detection of antimicrobial peptide biosynthetic gene in strain LM-1 by PCR. M: 2000 bp DNA marker; 1: *srf*; 2: *itu*; 3: *fen*. Figure S2: GO Annotated distribution bar chart. The horizontal axis was the secondary classification of GO, and the vertical axis was the number of genes in this classification (right) and its percentage of the total number of annotated genes (left). Different colors represent different ortholog. Figure S3: KEGG Pathway classification histogram. The vertical axis was the name of the metabolic pathway involved, and the vertical axis was the number of genes annotated to the pathway. Figure S4: The database annotates the rate line chart. The horizontal axis was the database name, and the vertical axis was the proportion of genes annotated to that database. Figure S5: Database annotated gene Venn map. Figure S6: Effect of *B. amyloliquefaciens* LM-1 on inhibition of *M. oryzae* and *B. oryzae*. (A) and (B): Pot spray inoculation experiment. *M. o*: *M. oryzae*, *B. o*: *B. oryzae. M. o +* LM-1: the strain LM-1treatment with *M. oryzae*. *B. o +* LM-1: the strain LM-1treatment with *B. oryzae.* Disease symptom of *M. oryzae* and *B. oryzae* were assessed at 7 days. Table S1: Primers used in this study. Dataset S1: Annotation statistics of *B. amyloliquefaciens* LM-1. Dataset S2: Gene prediction of *B. amyloliquefaciens* LM-1.
