# Supplementary material for: Bacillus amyloliquefaciens LM-1 Affects Multiple Cell Biological Processes in Magnaporthe oryzae to Suppress Rice Blast
Source: Microorganisms. 2024 Jun 20;12(6):1246. doi: 10.3390/microorganisms12061246 (PMC11205629; doi:10.3390/microorganisms12061246)
Supplement: Supplementary file 1 [file microorganisms-12-01246-s001.zip › Table S1 .pdf]

Table S1 Primers used in this study

Gene name	Primer sequence
Surfactin	5′-ATGAAGATTTACGGAATTTA-3′<br>5′-TTATAAAAGCTCTTCGTACG-3′
Iturin	5′-ATGTATACCAGTCAATTCC-3′<br>5′-GATCCGAAGCTGACAATAG-3′
Fengycin	5′-CTATAGTTTGTTGACGGCTC-3′<br>5′-CAGCACTGGTTCTTGTCGCA-3′
16S	27F 5′-AGAGTTTGATCCTGGCTCAG-3′<br>1492R 5′-GGTTACCTTGTTACGACTT-3′
