# Supplementary figures and images for: Bacillus amyloliquefaciens LM-1 Affects Multiple Cell Biological Processes in Magnaporthe oryzae to Suppress Rice Blast
Source: Microorganisms. 2024 Jun 20;12(6):1246. doi: 10.3390/microorganisms12061246 (PMC11205629; doi:10.3390/microorganisms12061246)

**A**

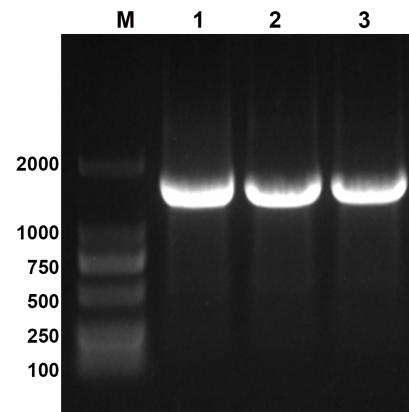

**B**

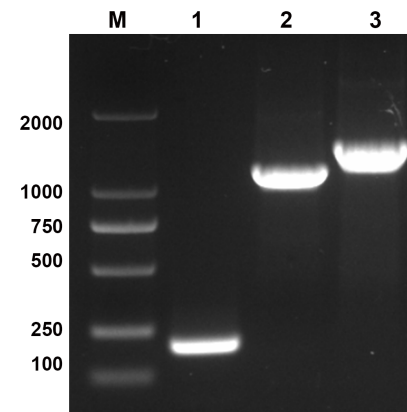

Supplement: Supplementary file 1 [file microorganisms-12-01246-s001.zip › Figure S1.pdf]

# GO Classification

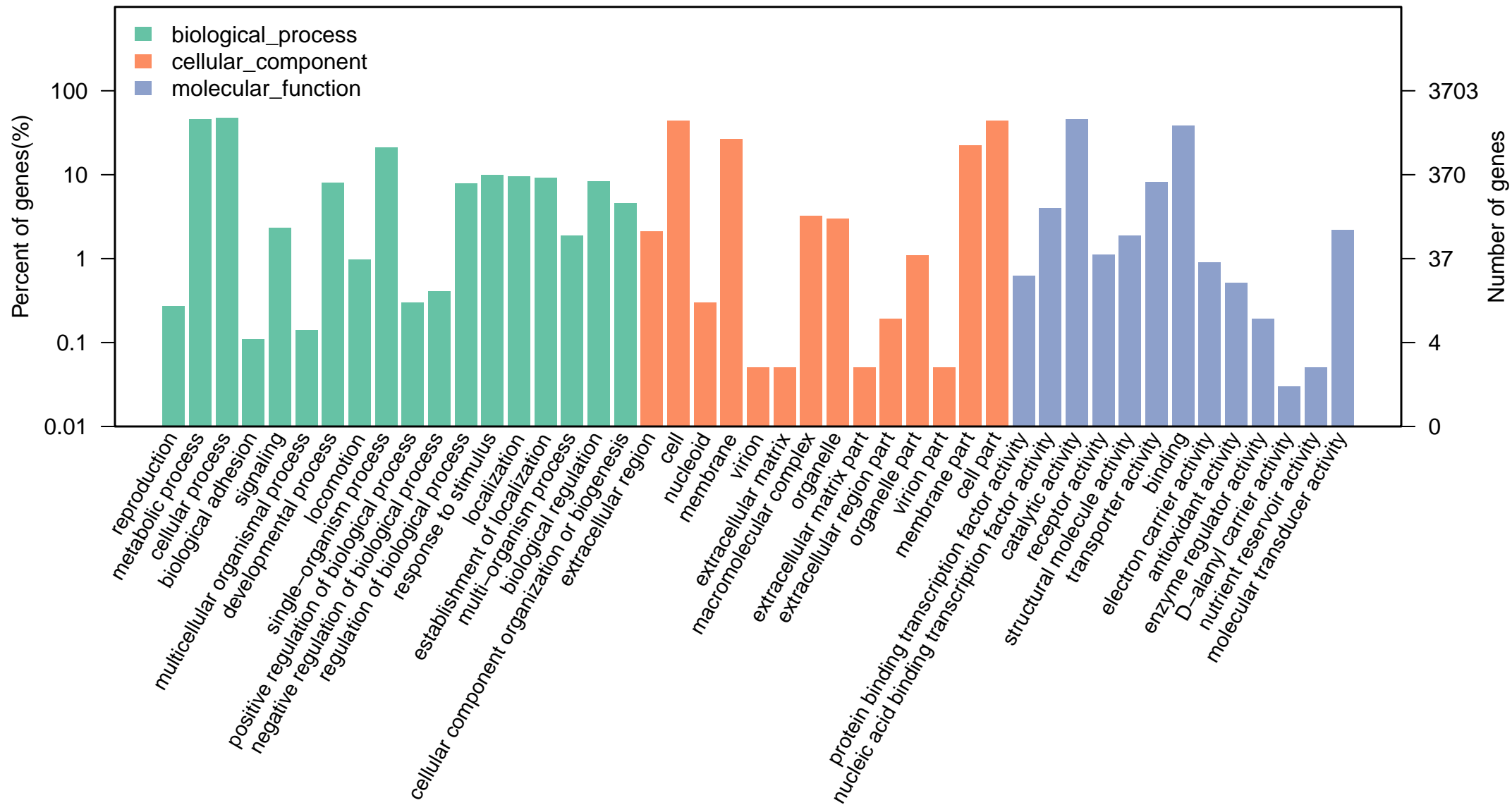

Supplement: Supplementary file 1 [file microorganisms-12-01246-s001.zip › Figure S2 .pdf]

# KEGG Classification

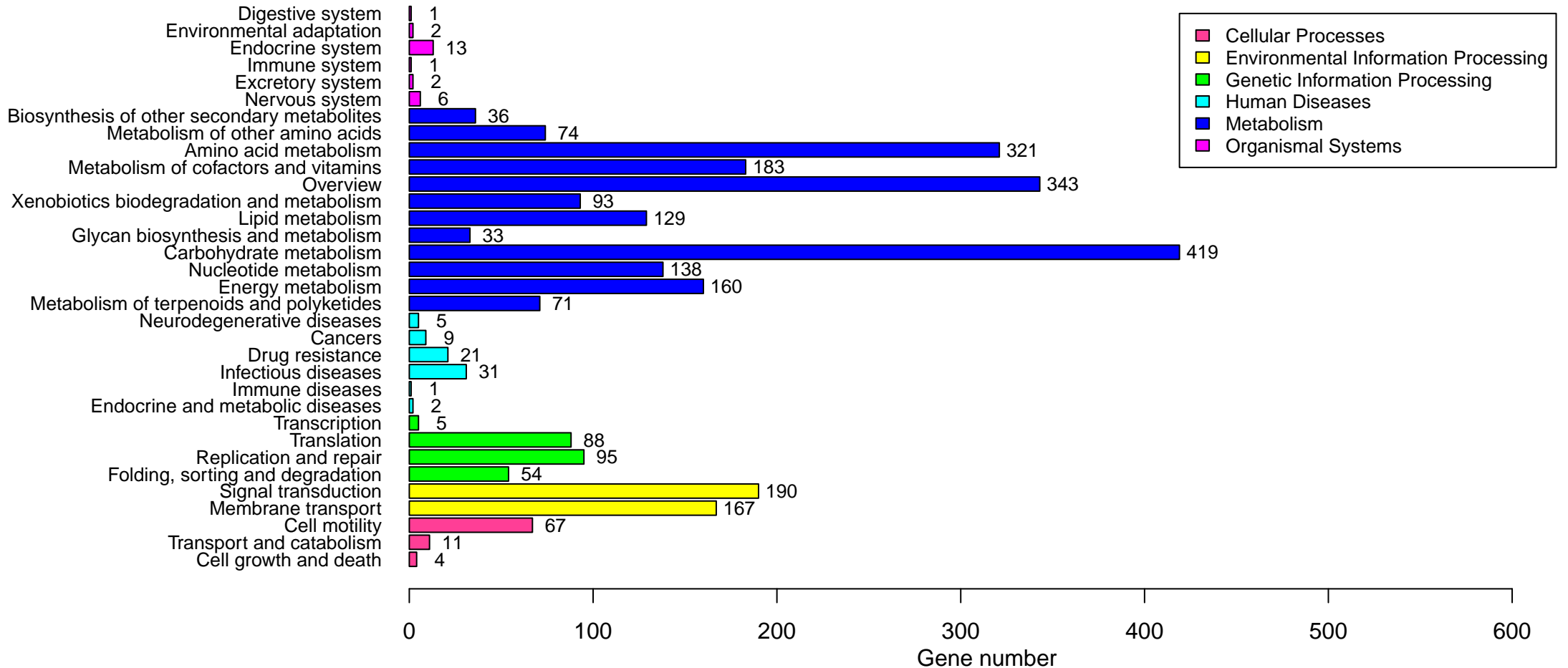

Supplement: Supplementary file 1 [file microorganisms-12-01246-s001.zip › Figure S3.pdf]

## Percentage of annotated genes

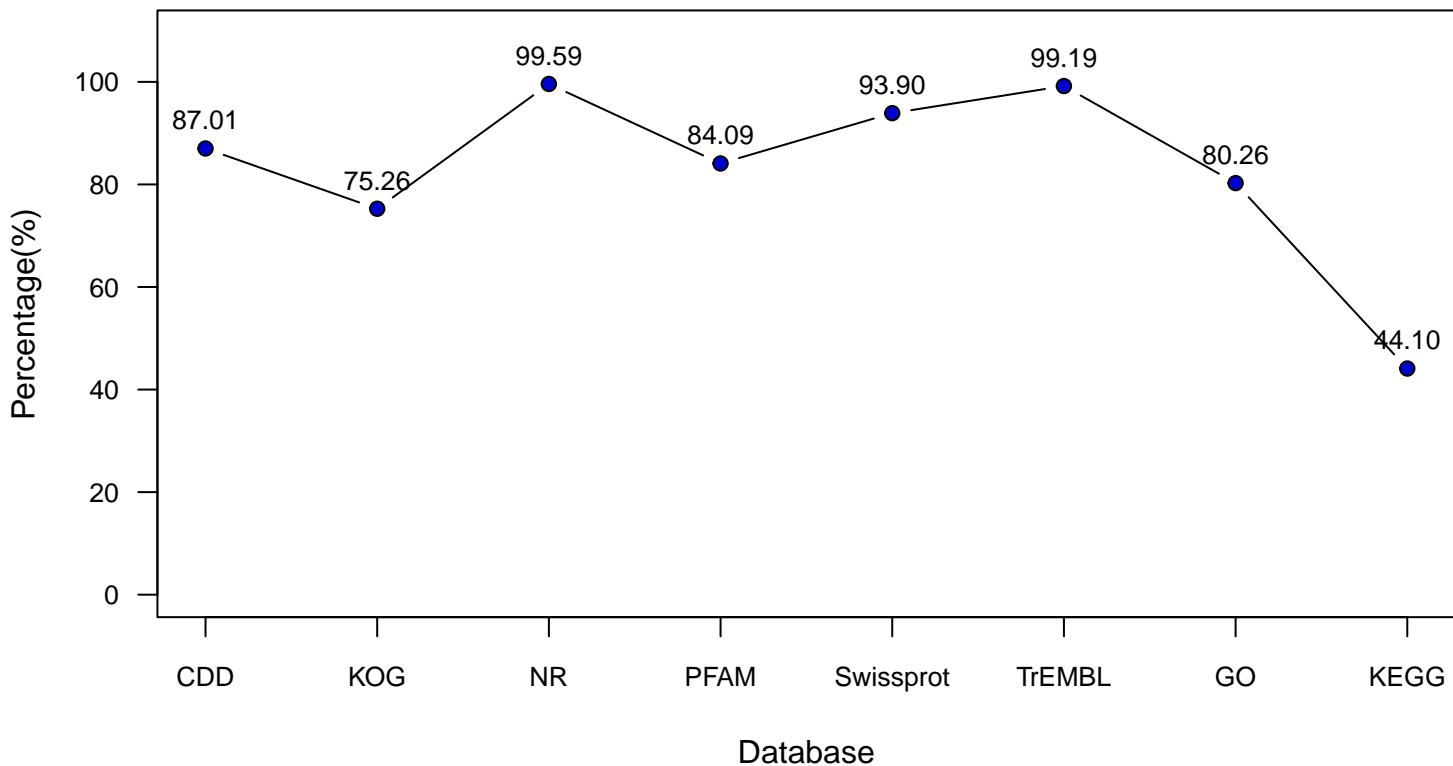

Supplement: Supplementary file 1 [file microorganisms-12-01246-s001.zip › Figure S4.pdf]

# Venn diagram for Database

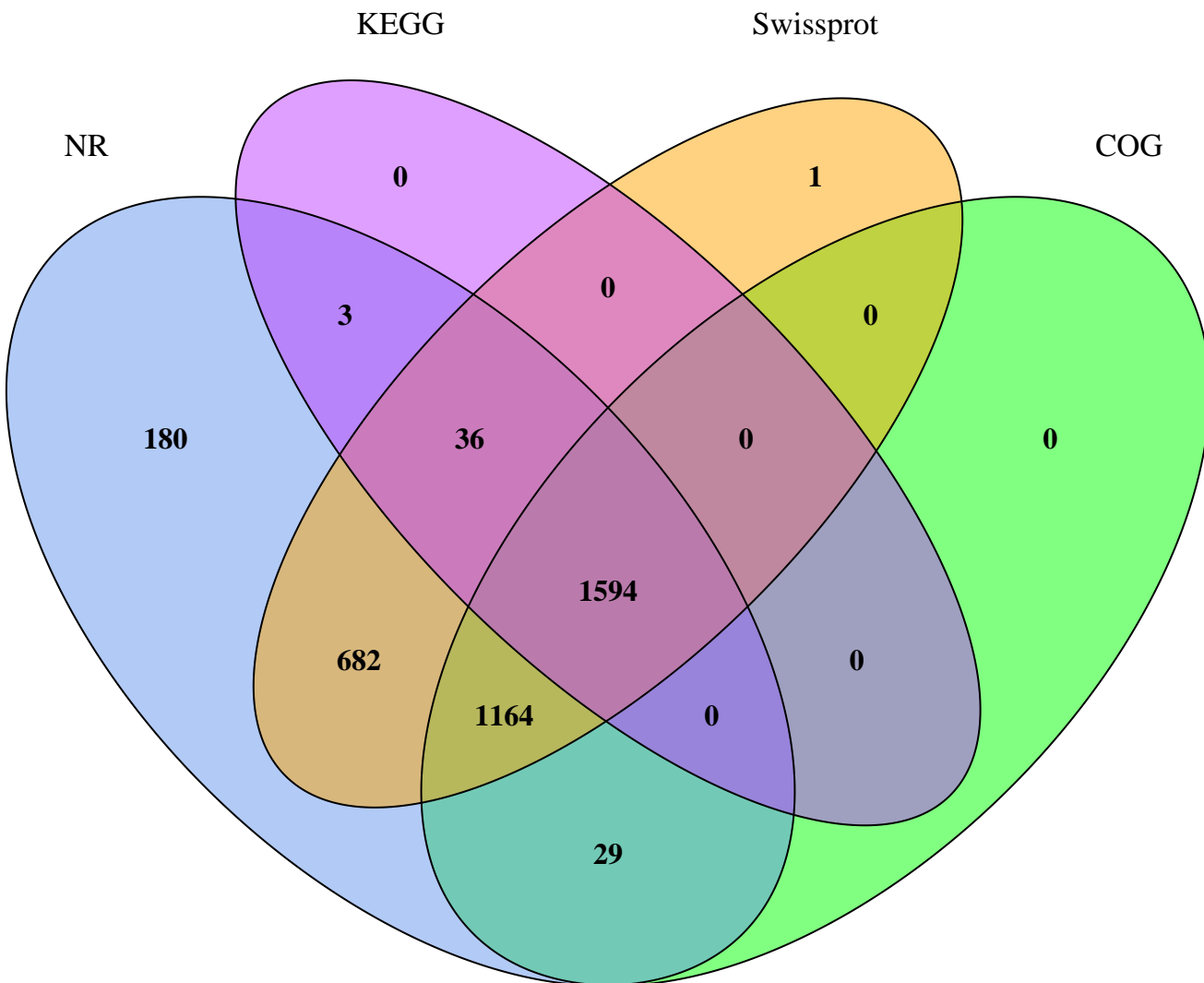

Supplement: Supplementary file 1 [file microorganisms-12-01246-s001.zip › Figure S5.pdf]

**A**

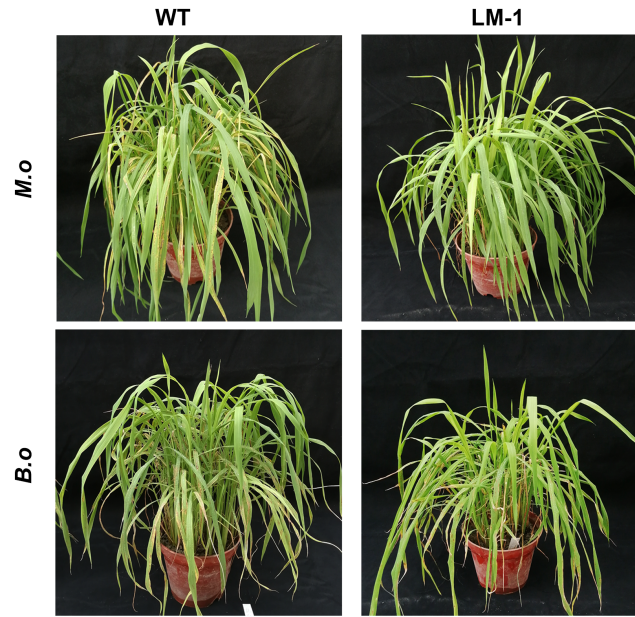

**B**

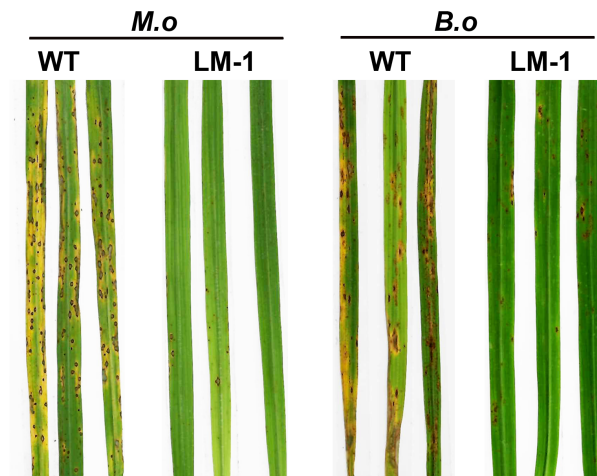

Supplement: Supplementary file 1 [file microorganisms-12-01246-s001.zip › Figure S6.pdf]
